# SEL1L Regulates Adhesion, Proliferation and Secretion of Insulin by Affecting Integrin Signaling

**DOI:** 10.1371/journal.pone.0079458

**Published:** 2013-11-20

**Authors:** Giuseppe R. Diaferia, Vincenzo Cirulli, Ida Biunno

**Affiliations:** 1 Integrated System Engineering, Milan, Italy; 2 Department of Medicine, University of Washington, Institute for Stem Cells and Regenerative Medicine, Seattle, Washington, United States of America; 3 Stem Cell Science Unit, IRCCS Multimedica, Milan, Italy; 4 Institute of Genetic and Biomedical Research (IRGB), National Research Council, Milan, Italy; Thomas Jefferson University, United States of America

## Abstract

SEL1L, a component of the endoplasmic reticulum associated degradation (ERAD) pathway, has been reported to regulate the (*i*) differentiation of the pancreatic endocrine and exocrine tissue during the second transition of mouse embryonic development, (*ii*) neural stem cell self-renewal and lineage commitment and (*iii*) cell cycle progression through regulation of genes related to cell-matrix interaction. Here we show that in the pancreas the expression of SEL1L is developmentally regulated, such that it is readily detected in developing islet cells and in nascent acinar clusters adjacent to basement membranes, and becomes progressively restricted to the islets of Langherans in post-natal life. This peculiar expression pattern and the presence of two inverse RGD motifs in the fibronectin type II domain of SEL1L protein indicate a possible interaction with cell adhesion molecules to regulate islets architecture. Co-immunoprecipitation studies revealed SEL1L and ß1-integrin interaction and, down-modulation of SEL1L in pancreatic ß-cells, negatively influences both cell adhesion on selected matrix components and cell proliferation likely due to altered ERK signaling. Furthermore, the absence of SEL1L protein strongly inhibits glucose-stimulated insulin secretion in isolated mouse pancreatic islets unveiling an important role of SEL1L in insulin trafficking. This phenotype can be rescued by the ectopic expression of the ß1-integrin subunit confirming the close interaction of these two proteins in regulating the cross-talk between extracellular matrix and insulin signalling to create a favourable micro-environment for ß-cell development and function.

## Introduction

*SEL1L* encodes an endoplasmic reticulum transmembrane protein with a complex structure implicated in a number of cellular functions [1]–[4] mostly associated with the endoplasmic reticulum associated degradation (ERAD) and unfolded protein response (UPR) pathways [5]–[7]. SEL1L established function is to complex with the E3 ligase HRD1 to regulate degradation and turnover of luminal and membrane proteins [8]. *SEL1L* is located close to the D14S67 locus on the chromosome 14q24 [9], [10] hypothesized to be a candidate region for the type I diabetes mellitus (T1DM) [11]. However no evidence for *SEL1L* as candidate gene for IDDM11 was found [12], [13]. Interestingly, it was suggested that mutations in *SEL1L* could influence MODY onset and/or progression [14]. To date, six MODY genes have been identified (glucokinase, hepatocyte nuclear factors *HNF-1α*, *HNF-4α* and *HNF-1β*, insulin promoter factors *IPF1* and *NEUROD1*), all associated with islet cells development, maintenance of differentiation, and endocrine secretory function [15]–[18]. Interestingly, HNF-1α and HNF-4α have been reported to bind the *SEL1L* promoter, supporting its involvement in pancreas development [19]. It was reported that mice homozygous for a gene trap mutation in *Sel1l* developed systemic ER stress and died during mid-gestation [20] like the *Hrd1* knock-out mouse model [21] but, in addition, *Sel1l* mutants displayed severe growth retardation and impaired differentiation of pancreatic and neural epithelial cells, suggesting an HRD1-indipendent function(s). Mice carrying one functional allele, revealed an increased susceptibility to diet-induced hyperglycemia and reduced β-cell mass [22], [23], and its depletion in βTC3 cells resulted in vitro growth arrest and cell death [24]. All together these results suggest that SEL1L could play a significant role in regulating ß-cell function and growth. To date, a number of mechanisms have being proposed to explain the progressive loss of β-cell function that eventually leads to T2DM. Among them, ER-stress responses induced by chronically elevated circulating levels of glucose and lipids, collectively known as glucolipotoxicity [25], are centain to have a detrimental impact on β-cell function, and possible β-cell death [26], [27]. More recently, evidence has been provided in support of more complex mechanisms of progressive impairement of β-cell function that involves a loss of β-cell identity rather than death by apoptosis, which leads to β-cell dedifferentiation into embryonic-like endocrine progenitors and interconversion into α-cell [28].

Causative mutations in *SEL1L* are very rare, however polymorphic variants have been reported: one associated with pancreatic cancer [29], a second with persistent hyperinsulinemic hypoglycemia of infancy [30] and a third in progressive childhood ataxia [31]. Of particular interest is the late evolutionary addition of the Fibronectin type II domain to the gene, increasing the protein functional complexity by contributing to cell-matrix interactions [32]. This domain is usually found in extracellular matrix fibronectin and in extra cytoplasmic regions of membrane associated-proteins and are thought to be involved in protein cell surface localization and activation through collagen-β1 integrin binding [33], [34]. Integrin engagement is a key regulator of pancreatic β-cell function, induces ERK-dependent insulin secretion and promotes epithelial to mesenchymal transition (EMT) by regulating the WNT/SMAD pathway [35]–[37]. More recently, β1 integrin-dependent signaling has also been implicated in the regulation of embryonic and perinatal ß-cell expansion [38]. Moreover, SEL1L has been reported to play a key role in the improvement of pancreatic plasticity being involved in the combined action of several pathways such as WNT, TGF-β, NOTCH and MAPK [39].

Here we show that SEL1L down-modulation in pancreatic β-cells negatively impacts on cell adhesion and proliferation, and inhibits glucose-stimulated insulin secretion by affecting ERK signaling. We also show that this phenotype can be rescued by overexpressing β1 integrin subunit and restoring ERK activation level.

Collectively, our results support a possible function of SEL1L in regulating the cross-talk between integrin signaling and insulin secretion.

## Materials and Methods

### Cell Lines, Culture Conditions and Transfections

CFPAC-1 human ductal adenocarcinoma cells (ATCC) were grown in Iscove’s modified Dulbecco’s medium (Life Technologies) supplemented with 10% fetal bovine serum and 2 mM L-Glutamine. MIN6 cells (obtained from Prof. Paolo Meda, University of Geneva, Switzerland [40] originally from Dr Miyazaki [41]) were grown in DMEM-high glucose medium with 2 g/L sodium bicarbonate, supplemented with 10% FBS and 70 µM of β-Mercaptoethanol.

Islets were isolated by intraductal injection of 0.5 mg/ml liberase and purified on a Ficoll gradient [42]. Islets were cultured overnight in RPMI-10% FCS and handpicked before being further processed.

MIN6 cells were transiently trasfected with 100 nM of siRNA against exon 3 of mSEL-1L or siRNA negative control (Applied BioSystems, Life Technologies), with or without 1 mg of β1-integrin expressing plasmid using Lipofectamine 2000 following the manufacturer instructions (Life Technologies). This construct was generated by subcloning the mouse full-length β1-integrin cDNA sequence, generated by PCR (using the following primers: ms Itgb1-BamHI: 5′-CCGGGATCCACCATGAATTTGCAACTGGTTTCC-3′; ms Itgb1-EcoRI: 5′- CCGGAATTCTCATCATTTTCCCTCATACTTCGG-3′) into pCDNA3.1(+) using BamHI/EcoRI sites. The construct was verified by Sanger sequencing.

Mouse islets were dissociated with 0.005% trypsin diluted in Versene (Life Technologies) lightly expanded and transiently nucleofected with 100pmoli of siRNA against exon 3 of mSEL-1L or siRNA negative control. Program T-020 of the Nucleofector® instrument (Lonza, Basel, Switzerland) and Reagent V kit (Lonza) were used following the manufacturer’s instructions. Nucleofected cells were plated onto HTB9-coated dishes [43]–[45] and cultured for 48 hours in RPMI-10% FCS medium.

### Imunofluorescence and Immunohistochemistry

Dual immuno fluorescent labeling was performed on 4-µm paraffin sections prepared from adult (3-month-old) and fetal (E16.5) of C57BL/6J mice pancreata. Use of animal subjects was carried out in strict accordance with the recommendations for the Care and Use of Laboratory Animals of the National Institute of Health (Italy). The protocol was approved by the Committee on the ethics of Animal Experiments of the National Research Council (Protocol Number Biunno-2/2011). All surgery was performed under Avertin anesthesia, and all efforts were made to minimize suffering. Human fetal pancreata (18–21 weeks gestational age) were obtained from Advanced BioResources (Alameda, CA) and samples of human adult pancreas were prepared at The Diabetes Research Institute (University of Florida, Miami, FL) as previously described [46]. Human pancreatic tissue specimens were provided as “preexisting pathological specimens” (i.e., not through the recruitment of living human subjects), with written consent for tissue donation obtained by the procurement entity. All studies described in this study were reviewed and approved by the Human research Protections Programs of the University of California San Diego (la Jolla, CA) and the University of Washington (Seattle, WA).

Tissues were fixed in 4% paraformaldehyde (PFA) and processed for paraffin embedding. After rehydratation in grades of ethanol, sections were boiled for 15 min in Citrate Buffer pH6.0. Cultured MIN6 cells were grown onto glass coverslips and fixed with 4%PFA for 20 min at 4°C, permeabilized with 0.1% Triton X-100 for 10 min and treated with 1 N HCl for 15 min at 65°C. After blocking, samples were incubated with primary antibodies at the following dilution: anti-insulin (1∶1000, The Bindind Site PC059.X), anti-glucagon (1∶100, Millipore AB932), anti-SEL1L (5 mg/ml) [47], anti-BrdU (1∶100, Sigma-Aldrich B8434) and anti-β1-integrin (10 mg/ml, Millipore MAB1997) and revealed with appropriate secondary antibodies (Rhodamine-Red anti-sheep IgG, anti-rat IgG and anti-rabbit IgG, Jackson Immuno Research, West Grove, PA, USA; Alexa Fluor 488 anti-mouse IgG, Molecular Probes, Invitrogen, Carlsbad, California, USA). Nuclei were counterstained with Hoechst 33258 and samples were mounted with GelMount aqueous mounting medium (SIGMA). Images were acquired using a LEICA DMI4000B inverted fluorescence microscope linked to a DFC360FX camera (LEICA Microsystems, Vienna, Austria).

### Cell Adhesion, Proliferation and Secretion Assay

For adhesion assay, MIN6 trasfected cells and untreated control were plated onto different ECMs as previously described [48]. After 1 hour, cells were fixed in 4%PFA, stained with 1% Tolouidine and counted under the microscope.

For *in vitro* proliferation assessment, MIN6 trasfected cells and untreated control were pulsed with 10 µM BrdU (Sigma-Aldrich) and cultured for 1 hour. Cells were then fixed and processed for immunofluorescence analysis as described below. BrdU-positive cells were counted in 5 random fields under a fluorescence microscope and results expressed as percentage of total counterstained nuclei. For *in vitro* insulin secretion measurements, MIN6 transfected and mouse islet nucleofected cells were incubated in HEPES-balanced Krebs-Ringer buffer (KRBH), pH 7.4 (10 mM HEPES, 120 mM NaCl, 4.7 mM KCl, 1.2 mM MgCl2, 1.2 mM NaH2PO4, 25 mM NaHCO3, 2.5 mM CaCl2) supplemented with 0.25% bovine serum albumin and deprived of glucose for 1 hour and then 2.8 mM glucose was added for a further hour. Cells were washed and the medium was collected after 30-min incubation with fresh KRBH solution containing 2.8 mM glucose followed by 1-hour stimulation with 22.8 mM glucose. Insulin secreted in the medium was measured by ultrasensitive EIA (Alpco Diagnostic); results were normalized by DNA content determined with Quant-IT PicoGreen reagent and Qubit fluorimeter (Life Technologies).

### Co-immunoprecipitation and Western Blot

For co-immunoprecipitation studies, CFPAC-1 cells were lysed in 20 mM Tris-HCl (pH 7.5), 150 mM NaCl, 1 mM MgCl2, 2 mM EGTA, 10% glycerol and 1% NP40 in the presence of protease inhibitors. The lysates were clarified by centrifugation and total proteins were measured using the BCA protein assay (Pierce). Proteins (∼5 µg) were incubated with 5 µg of mAbs to β1-integrin (Biogenex), β4-integrin (Ancell Corp, 325-020), α6-integrin (Millipore, MAB1378) or 5 µl of pAbs to SEL1L (kindly provided by Prof H. Ploegh, [49]), α5-integrin (Millipore, AB1949) or α6-integrin (Millipore, AB1920) and incubated at 4°C overnight. Corresponding amount of control IgG and normal rabbit serum were used as negative controls. Immuno-complexes were captured with protein A/G agarose beads (Calbiochem) for 2 hours at 4°C, eluted from the beads by boiling in reducing Laemmli buffer and resolved on a 9% SDS-PAGE gel. Proteins were transferred to a PVDF membrane, blocked and incubated with 2 µg/ml mouse antibody to SEL1L [47] or mouse antibody to β1-integrin (1∶2500; Cell signaling).

For expression studies, MIN6 cells were lysed in RIPA buffer containing protease inhibitors (Roche), 1 mM PMSF, 1 mM EDTA, 1 mM sodium fluoride. Cell extracts were resolved on 9% SDS-polyacrilamide gel, blotted onto PVDF membranes, and probed with anti-SEL1L, anti-ß1-integrin, anti-ERK1/2 (1∶1000; Cell Signaling) anti-phospho ERK1/2 (1∶1000; Cell Signaling). Hybridizations were performed in sealed bags with X-blot-100 chamber (www.isenet.it), and proteins were detected with appropriate HRP conjugated secondary antibodies (Jackson Immuno Research) and the ECL system (Thermo Fisher Scientific).

### Q-PCR

Total RNA was purified from MIN6 and mous islet cells using Tri-Reagent (Applied BioSystems) according to manufacturer’s instructions and retro-transcribed with RevertAid cDNA synthesis kit (Thermo Scientific). SYBR green qPCR was performed on RotorGene Q (QIAGEN) machine, using Maxima SYBR green master mix (Thermo Scientific) and the following primers:

*Sel1l*: 5′-GCCCGATGAAGTGGAAAAC-3′; 5′-CATTCTTAAACAACTCCACTGC-3′, *Ccdn1*∶5′-TCGTGGCCTCTAAGATGAAGGA-3′; 5′- CCTCGGGCCGGATAGAGTT-3′ [50], *p21*∶5′-TTGTCGCTGTCTTGCACTCT-3′; 5′- AATCTGTCAGGCTGGTCTGC-3′, *Hprt*: 5′-GTGCTCAAGGGGGGCTATAA-3′; 5′- GGTCCTTTTCACCAGCAAGC-3′. Data were normalized to *Hprt* expression using ΔΔCt method.

### Statistics

Statistical significance of differences in data values was validated by two tailed student’s *t* test with significance limit set at p<0.05.

## Results

### Immunofluorescence (IF) Analysis of SEL1L Expression in Fetal and Adult Pancreas

**Figure 1:**
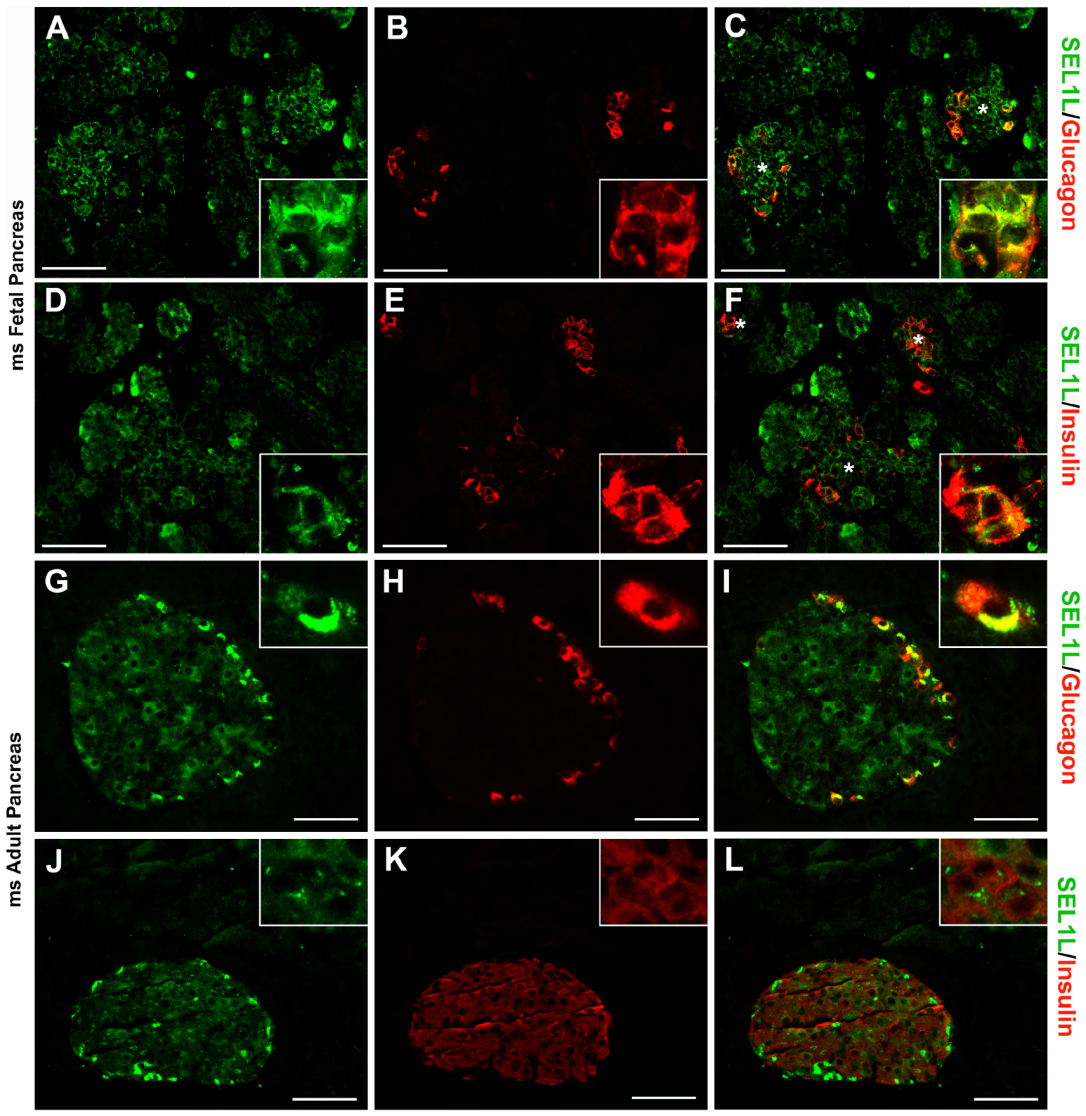
SEL1L expression in fetal and adult mouse pancreas. Representative images of pancreatic sections from E16.5 mouse embryos (**A**–**F**) and 8-weeks-old mice (**G**–**L**) immunostained for SEL1L (green; **A**, **D**, **G** and **J**), glucagon (red; **B** and **H**) and insulin (red; **E** and **K**). Dual-color immunoflurescence showed SEL1L specific immunoreactivity (*green,* **C** *and* **F**) in the nascent acinar tissue and in the developing islets (*asterisks*) stained for glucagon (red, **C**) and insulin (*red,* **F**). While exocrine tissue, in the adult mouse, didn’t show any SEL1L immunoreactivity (*green,* **I** *and* **J**), endocrine cells revealed a marked expression of SEL1L protein with a strong cytoplasmic immunoreactivity in α-cells (stained for glucagon in red, **I**) and a moderate expression in β-cells (stained for insulin in red, **L**). Scale bar = 50 µm.

**Figure 2:**
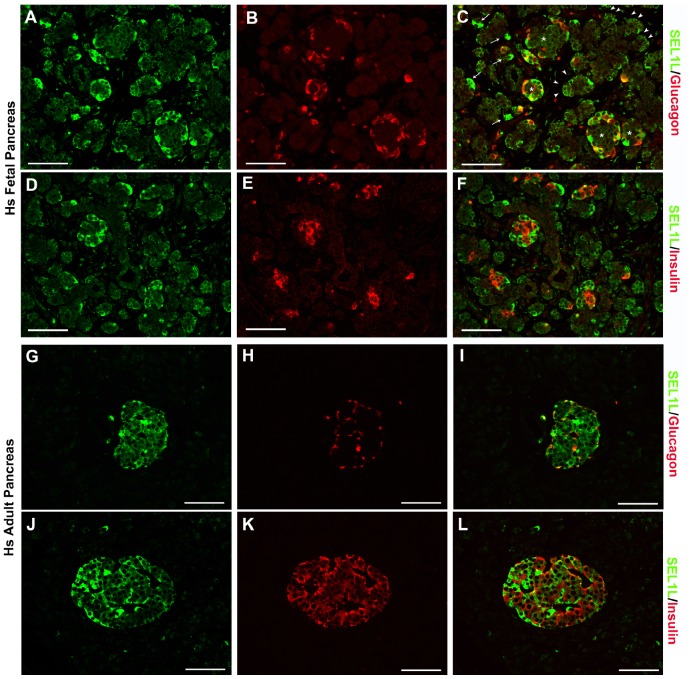
SEL1L expression in fetal and adult human pancreas. Representative images of pancreatic sections from 18 weeks fetus (**A**–**F**) and 54-years-old patient (**G**–**L**) immunostained for SEL1L (green; **A**, **D**, **G** and **J**), glucagon (red; **B** and **H**) and insulin (red; **E** and **K**). Dual-color immunoflurescence showed SEL1L specific immunoreactivity (*green,* **C** *and* **F**) in the nascent acinar cells adjacent to basement membrane (*arrowheads*) and in few interspersed cells (*arrows*); a strong cytoplasmatic staining is also observed in the developing islets (*asterisks*) stained for glucagon (red, **C**) and insulin (*red,* **F**); in adult human pancreas, the exocrine tissue didn’t show any SEL1L immunoreactivity (*green,* **G** *and* **J**), while endocrine cells revealed a marked expression of SEL1L protein with a strong cytoplasmic immunoreactivity in α-cells (stained for glucagon in red, **I**) and β-cells (stained for insulin in red, **L**). Scale bar = 50 µm.

IF analysis of murine fetal (E16.5) and adult pancreas identifies SEL1L-specific immunoreactivity in developing acinar and in α- and β-cells (Fig. 1A–F). Interestingly, as development proceeds, SEL1L expression becomes restricted to the α- and β-cells (Fig. 1 G–L), virtually absent in the acini, with only occasional single cells dispersed throughout the entire organ. Among the different types of islet cells, SEL1L-specific immunoreactivity is significantly stronger in the cytoplasm of α-cells, compared to β-cells (Fig. 1G–L). This expression pattern appears conserved in the human pancreas (Fig. 2A–L), with strong immunoreactivity detected in developing islets, nascent acinar clusters (Fig. 2A–F) and in differentiating islet α- and β-cells (Fig. 2G–L). Interestingly, both in mouse and human pancreas, SEL1L immunoreactivity localizes preferentially to cells adjacent to the basement membranes and vascular/ductal structures, suggesting a possible interaction with the extracellular matrix (ECM) (Figure S1). Collectively, these results indicate the existence of a spatiotemporal regulation of SEL1L expression during pancreas development, and suggest important functions in the islet cell compartment, possibly involving the regulation of hormone secretory function.

### SEL1L Co-immunoprecipates with β1 Integrin

**Figure 3:**
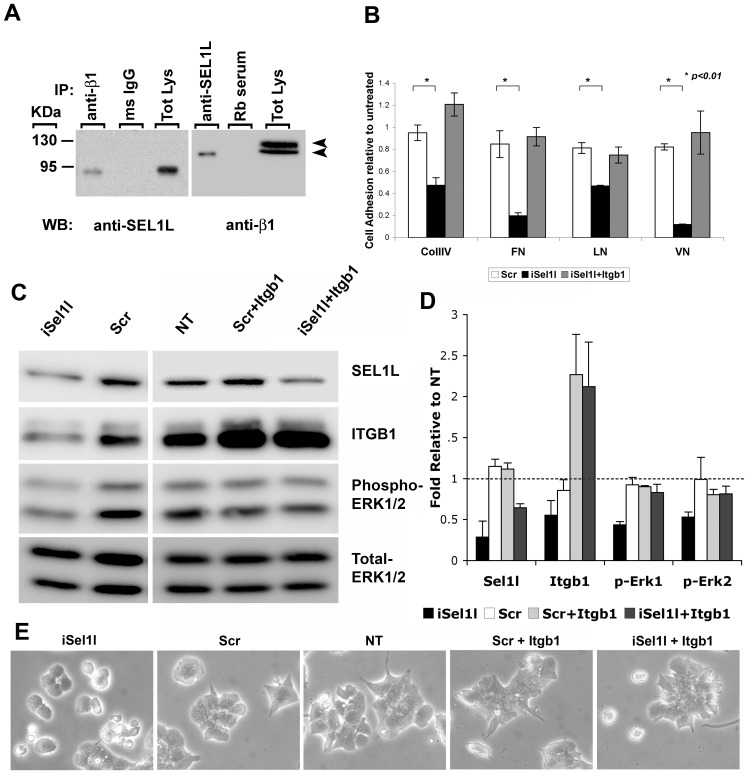
SEL1L interacts with integrins. (**A**) Co-immunoprecipitation for integrin subunits followed by western blotting for SEL1L (*left panel*) and viceversa (*right panel*); arrows indicate 130-kDa mature β1 integrin and 110-kDa precursor. (**B**) MIN6 cells immunostained for SEL1L (*green*) and β1 integrin (*red*); a higher magnification of SEL1L/ß1 integrin co-localization to plasma membranes is shown in the inset of the right panel. Nuclei (*blue*) are counterstained with Hoechst 33258.

The presence of the Fibronectin type II domain (a collagen binding domain) in SEL1L exon 4 [1], [30] led us to postulate the possible interaction of SEL1L with integrin receptors. To investigate this possibility, we used the human pancreatic ductal cell line CFPAC-1, a valuable model for assessing pancreatic cell adhesion and migration on selected ECMs [51], [52], and tested SEL1L ability to interact with integrin receptor subunits by classical co-immunoprecipitation experiments. Due to lack of reliable antibodies to mouse SEL1L for IP assays, and lack of human pancreatic β-cell line, we decided to use the human CFPAC-1 cell line only for co-IP experiments with integrins, and the mouse β-cell line MIN6 for all of the functional studies. Therefore, CFPAC-1 lysate were immunoprecipitated with anti-integrin antibodies, resolved by SDS-PAGE, and probed with SEL1L antibody. As shown in Figure 3A, α5-, β1-, β4- integrin-specific antibodies co-immunoprecipitated SEL1L. Interestingly among the a subunits only α5 integrin is able to co-immunoprecipate with SEL1L, while among the β subunits, β1-integrin is able to pull-down SEL1L more efficiently than β4, suggesting that the fibronectin receptor α5β1 is the main integrin heterodimer able to interact with SEL1L. We therefore focused on the β1-integrin subunit to test the reverse approach. Interestingly, SEL1L-specific antibody co-immunoprecipitated the 110-kDa β1 precursor, but not the mature 130-kDa form, suggesting that SEL1L may interact with a protein complex prior to the post-translational modification of the β1 integrin-subunit to mediate the recruitment and assembly of integrin αβ heterodimer receptors. However, it remains to be determined whether this interaction occurs directly, i.e. β1 integrin/SEL1L, or indirectly through an alternative binding mechanism such as recruitment into a β1 integrin/Collagen/SEL1L.

### SEL1L Downmodulation Interferes with Integrin-mediated Adhesion and ERK Activation in MIN6 Cells

**Figure 4:**
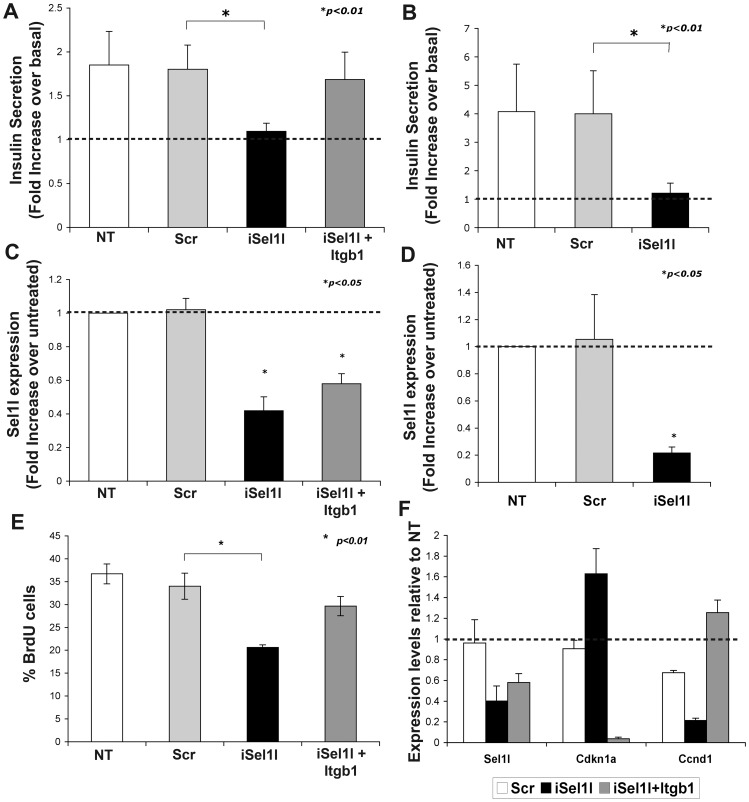
SEL1L affects cell adhesion. (**A**) MIN6 cells were transfected with: scrambled-control (*white bars*), siRNA against *Sel1l* (*black bars*) or co-transfected with *Sel1l-*siRNA and β1 integrin over-expressing construct (*grey bars*) were left to adhere for 1 hour on 96-well plate coated with Collagen-IV (*CollIV*), Fibronectin (*FN*), Laminin (*LN*) and Vitronectin (*VN*). Adherent cells were then fixed, stained and counted under a microscope. Values are relative to untransfected adherent cell and presented as means ± SD from three independent experiments. (**B**) Western blot analysis of MIN6 cells (NT) transfected with scramble-control (*Scr*), with siRNA against *Sel1l* (*iSel1l*) and co-transfected with β1 integrin over-expressing construct (*Scr+Itgb1*and *iSel1l+Itgb1*) probed for SEL1L, β1 integrin (ITGB1), phospho-ERK1/2 and total-ERK1//2. (**C**) Quantification of the immunoblot bands; values are representative of three independent experiments and are expressed as fold of expression ± SD relative to NT and normalized to total ERK1/2 as loading control. (**D**) Photomicrographs showing the effect of SEL1L down-modulation on MIN6 morphology; the *Sel1l*-interfered cells (*iSel1l*) appears round-shaped compared to controls (*Scr, NT, Scr+Itgb1*) while overexpression of β1 integrin subunit restore MIN6 polygonal spindle-like morphology (*iSel1l+Itgb1*).

The functional relationship between SEL1L and β1 integrin was analyzed in MIN6 cells. These cells have extensively been used as a reliable model system to study the biology of pancreatic β-cells since they retain a high degree of endocrine differentiation (i.e. insulin expression) and glucose-stimulated insulin secretion [41]. Hence, we first confirmed the colocalization of SEL1L and β1-integrin in MIN6 cells. Immunofluorescence analysis revealed SEL1L and β1-integrin specific immunoreactivity targeted to plasma membrane, suggesting a functional activity in “inside-out” integrin signaling and/or trafficking of the integrin receptors (Fig. 3B). MIN6 cells were transfected with either siRNA specific for *Sel1l* transcripts, or with a scrambled control, and then used in cell adhesion short-term assays to study their ability to adhere to a panel of ECM proteins (Collagen-IV, Fibronectin, Laminin and Vitronectin) known to promote β-cell attachment and function. *Sel1l*-interfered cells adhered less efficiently to the matrixes tested when compared to the mock-transfected ones (Fig. 4A). Interestingly, overexpression of the β1 integrin subunit in *Sel1l*-interfered cells rescued their adhesion defect to all matrixes analyzed, suggesting that constitutive β1 integrin overexpression is able to override its dependence on SEL1L. In fact, SEL1L-knockdown appears to efficiently down modulate β1 integrin protein levels resulting in impaired utilization of endogenous levels of β1 integrin and reduced activation of ERK1/2 (phospho ERK1/2) (Fig. 4B–C). Furthermore, interfered cells lost the usual polygonal shape and assumed a round-shape morphology (Fig. 4D), suggesting a switch from cell-matrix to cell-cell adhesion mechanisms as a consequence of a reduced adherence to ECMs. Overexpression of β1 integrin construct restored ERK activation (Fig. 4B) and the cells re-acquired their normal morphological appearance (Fig. 4D). Cells transfected with control siRNA-scrambled alone or co-transfected with β1 integrin overexpressing construct did not significantly alter ERK activation levels, nor cell morphology, further attesting a significant functional interdependence between SEL1L and β1 integrin.

### SEL1L Downregulation Impairs Glucose-stimulated Insulin Secretion

**Figure 5:**
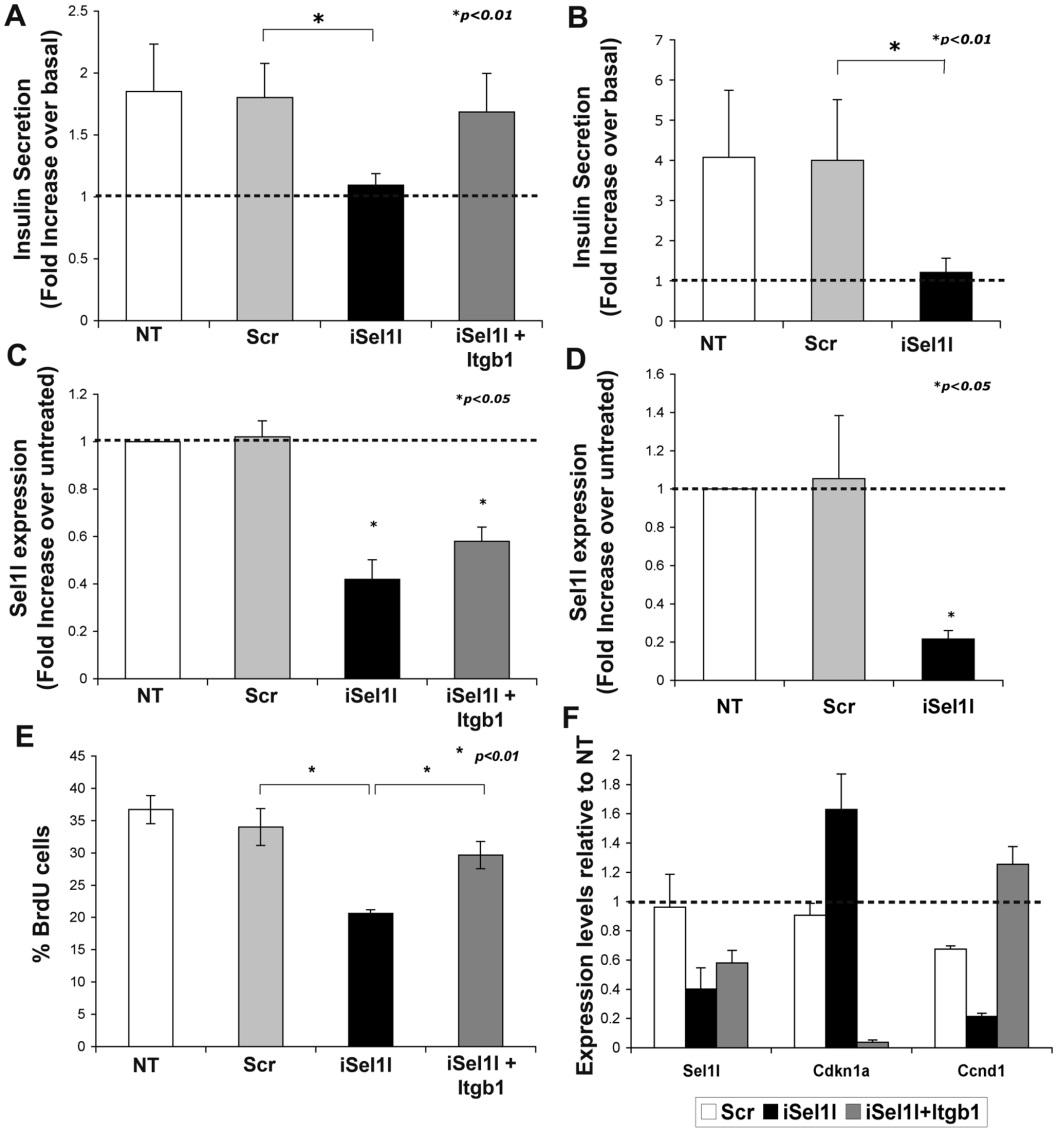
Impact of SEL1L down-modulation on insulin secretion and cell proliferation. Insulin release of MIN6 transfected (**A**) and mouse islet nucleofected (**B**) cells was quantified after 1-hour stimulation with 22.8 mM glucose. Values are normalized by DNA content and are expressed as a mean ± SD of fold increase in insulin release over basal glucose concentration from three separate experiments. MIN6 transfected cells (**C**) and mouse islet nucleofected cells (**D**) were assayed by qPCR for effective *Sel1l* down-modulation by siRNA. Values are expressed as fold expression ± SD relative to un-treated sample (value = 1) and normalized to *Hprt*. (**E**) To assess SEL1L-dependent cell proliferation, MIN6 transfected and untransfected cells were pulsed for 1 hour with BrdU, fixed and immunostained. Frequency of BrdU-positive cells were represented as percentage of total number of nuclei counted and expressed as means ± SD from three independent experiments. (**F**) Changes in the expression of the key regulators of the cell cycle progression Cyclin D1 (*Ccdn1*) and p21 (*Cdkn1a*) were validated by qPCR; values are expressed as fold expression relative to un-treated sample (value = 1) and normalized to *Hprt*.

Integrin heterodimers containing the β1 subunit have been implicated in the regulation of insulin secretion through ERK signaling, thus supporting an important function for this class of integrins in the secretory function of pancreatic β-cells [36], [37]. Therefore, we set out to determine if SEL1L down modulation interfered with the β-cells response to glucose. For these studies, MIN6 cells were first transfected with siRNA against *Sel1l* and, 48 hours later, used in assays of glucose-mediated insulin secretion. As shown in Figure 5A, *Sel1l*-interfered cells failed to respond to glucose stimulation at the maximal concentration of 22.8 mml/l, whereas a 2-fold increase over basal was observed in scrambled and un-treated control. Moreover, as predicted, overexpression of β1 integrin subunit restored the glucose responsiveness in anti-*Sel1l*-siRNA transfected cells.

Significantly, as shown in Figure 5B, these results were validated using isolated mouse islet cells nucleofected with *Sel1l* siRNA and grown for 48 hours onto HTB9 matrix coated dish [45]. Quantitative PCR performed on all samples confirmed the specific down regulation of *Sel1l* transcript over the control cells (Fig. 5C–D).

### SEL1L Donwmodulation Affects Proliferation of MIN6 Cells

SEL1L has been previously described to regulate cell cycle progression [53], [54] and β-cell proliferation [24] but little is known about how it can exert this function. This prompted us to investigate if this mechanism could be mediated by β1 integrin. MIN6 cells were transfected with siRNA against *Sel1l,* and proliferating cells were pulsed for 1-hour with BrdU. As shown in Figure 5E, *Sel1l*-interfered cells displayed about 50% reduction in the percentage of BrdU positive cells compared to the scramble-transfected control, or untreated cells. Overexpression of β1 integrin in *Sel1l*-interfered cells restored DNA synthesis in MIN6 cells. To confirm the involvement of SEL1L in the β1 integrin signaling, changes in levels of expression of the key players of the cell cycle progression mediated by integrins were analyzed by qPCR. As shown in Figure 5F, *Sel1l*-interfered cells displayed a significant down-regulation of the cyclin D1 (*Ccnd1*), and an up-regulation of cyclin inhibitor p21 (*Cdkn1a*), while overexpression of β1 integrin in *Sel1l*-interfered cells restored cyclin D1 level and strongly inhibit p21 expression. Collectively, these results demonstrate that SEL1L plays multiple functions in pancreatic β-cells, spanning from control of cell-matrix adhesion, to cell proliferation and insulin secretion.

## Discussion

Despite significant efforts devoted to the identification of genes regulating β-cell ontogeny in either animal models of pancreas development or in population of putative stem/progenitor cells, our knowledge of mechanisms regulating β-cell replication and/or regeneration remains relatively incomplete. In this report we present data supporting an important involvement of SEL1L in islet cell adhesion through modulation of β1 integrin signaling, insulin secretion, and replication. Hence, we find that SEL1L is highly expressed in fetal and adult islet clusters, and in developing acinar cells, with a subcellular localization in the basal pole of islet cells adjacent to the basement membrane and vascular/ductal structures, where β1 integrin-specific immunoreactivity is preferentially identified (Figure S1) [38], that is suggestive for a functional involvement of SEL1L in integrin signaling and/or trafficking of the integrin receptors. It has been reported that SEL1L is highly and predominantly involved in the quality control system managing UPR-inducing stresses [2], [55] and studies on mouse models have shown that haplo-insufficiency of SEL1L predisposes mice to high fat-induced hyperglycemia probably due to elevated ER stress and up-regulation of the unfolded protein response pathway. Loss of β-cell function in T2DM has been associated to endoplasmic reticulum stress responses induced by chronically elevated circulating levels of glucose and lipids, collectively referred to as glucolipotoxicity [25], [27]. However, how ER stress mechanistically impacts on β-cell replication and insulin secretion remains unclear [22].

Our data demonstrate that SEL1L is able to regulate cell adhesion, proliferation and secretion of β-cells through β1 integrin signaling. Both integrins and the extracellular matrixes are known to play an essential role in promoting β-cell migration, proliferation and differentiation through the activation of the ERK pathway. Our experiments of SEL1L knockdown uncovered a mechanistic link with β1 integrin-dependent adhesion and proliferation, possibly by a decreased activation of ERK signaling. Thus, SEL1L, as a key regulator of the ERAD pathway, may play an important role in the trafficking/folding of β1 integrin, such that its downregulation may lead to improper degradation of this adhesion receptor and a defective activation of downstream ERK signaling that can only be rescued by the ectopic overexpression of β1 integrin.

Although the direct interaction between SEL1L and β1 integrin remains to be demonstrated, it is likely to occur through the fibronectin type II domain within SEL1L. This domain is missing from the invertebrate orthologs of SEL1L, and arises only in the chordate lineage, indicating the acquisition of new functions in higher organisms. It is worth mentioning that among the chordates, only the mouse and rat SEL1L proteins show a variant form consisting in the absence of the FNII domain, likely due to alternative splicing (NP_035474 and NP_808794, respectively).

Interestingly, the SEL1L-FNII domain contains two inverse RGD motifs (DGR), a non-canonical adhesion sequence capable of binding to various RGD-dependent integrins, albeit with a low affinity [56]. DGR-containing peptides have been shown to compete with adhesive proteins for integrin interaction [56]–[59], and found to be important for the cell adhesion and biological activities mediated by basic fibroblast growth factor (FGF2) and secreted frizzled-related protein (sFRP) through modulation of Wnt signaling [60], [61]. Based on the well-known role of these pathways in the control of cell growth and differentiation, DGR containing proteins such as SEL1L may contribute to regulate the crosstalk between Wnt, integrin, and growth factor signaling. Further studies will be required to identify protein domains mediating SEL1L–/β1-integrin interaction.

SEL1L may also be involved in the regulation of glucose-induced insulin secretion. Thus, this is a complex function that requires a multifactorial regulations: (*i*) insulin needs to be properly folded and packaged in secretory granules before being shuttled to the plasma membrane, (*ii*) the right outside-in integrin-signaling cascade must be properly enacted, and (*iii*) glucose sensing mechanisms efficiently activated. In this complex cascade of events, SEL1L may control both the “inside-out” and “outside-in” trafficking of molecules important in islet β-cell function such as folding of insulin, integrin-transduction cascades from the microenviroment, and turnover of glucose transporters. Matrix interaction can also influence insulin secretion via engagement of β1 integrin, and alteration in the glycosylation levels of the glucose transporter GLUT2 can impair glucose-stimulated insulin release [62]. Our findings on SEL1L-dependent insulin secretion are consistent with prior reports linking SEL1L deficiency to severe impairment of protein secretory pathways [23]. In this context, our demonstration, that overexpression of β1 integrin rescues the insulin secretory defect resulting from SEL1L knockdown, further support an important role of SEL1L in the integration of extracellular cues with the intracellular machinery regulating endocrine secretory functions.

Collectively, our studies provide new insights on the implication of SEL1L in pancreatic islet cell development and function that involve the recruitment and interaction with cell adhesion receptors of the integrin family. Further exploration of SEL1L function in the integration of mechanism governing β-cell replication and function may have significant implications for the development of cell-based replacement therapies for diabetes.

## Supporting Information

Figure S1 (DOCX)
