# Correction: SEL1L Regulates Adhesion, Proliferation and Secretion of Insulin by Affecting Integrin Signaling

**DOI:** 10.1371/annotation/dfd1ec6c-79d1-4d02-9e0f-b1fed3a54508

**Published:** 2013-12-31

**Authors:** Giuseppe R. Diaferia, Vincenzo Cirulli, Ida Biunno

Figure 3 is an incorrect version of Figure 4 and the current Figure 4 is a duplicate of Figure 5. Please see the correct versions of Figure 3 and 4 here:

Figure 3: 

**Figure:**
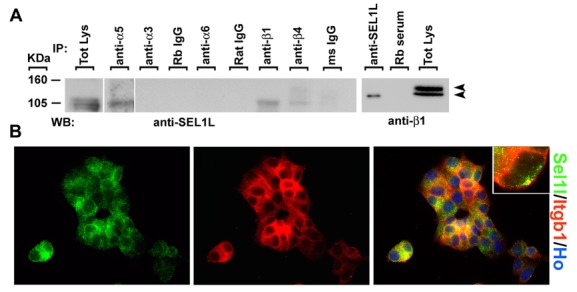


Figure 4: 

**Figure:**
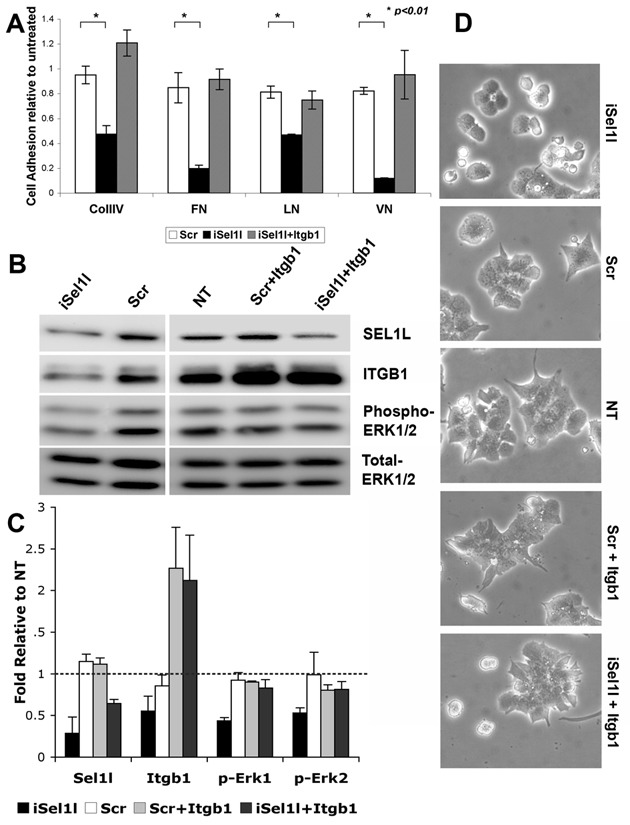


There was an error in the Funding section. The correct funding information is as follows: This work was supported by funds from Ministero Istruzione Universita`e Ricerca, FIRB:RBIPO64CRT-005. G. R. Diaferia was the recipient of a Postdoctoral fellowship from the Juvenile Diabetes Research Foundation. V. Cirulli was supported by an American Diabetes Association Basic Research Award (#1-11-BS-28) and Juvenile Diabetes Research Foundation Research Grants #1-2005-1084 and #17-2011-620, and an LSDF Program Project grant. The funders had no role in study design, data collection and analysis, decision to publish, or preparation of the manuscript.

There was an error in the Competing Interests section. The correct Competing Interests statement is as follows: The authors have the following interests: Giuseppe R. Diaferia was employed for a limited period of time by Integrated System Engineering. There are no patents, products in development or marketed products to declare. This does not alter the authors’ adherence to all the PLOS ONE policies on sharing data and materials, as detailed online in the guide for authors.

