# Supplementary figures and images for: SEL1L Regulates Adhesion, Proliferation and Secretion of Insulin by Affecting Integrin Signaling
Source: PLoS One. 2013 Nov 20;8(11):e79458. doi: 10.1371/journal.pone.0079458 (PMC3854660; doi:10.1371/journal.pone.0079458)

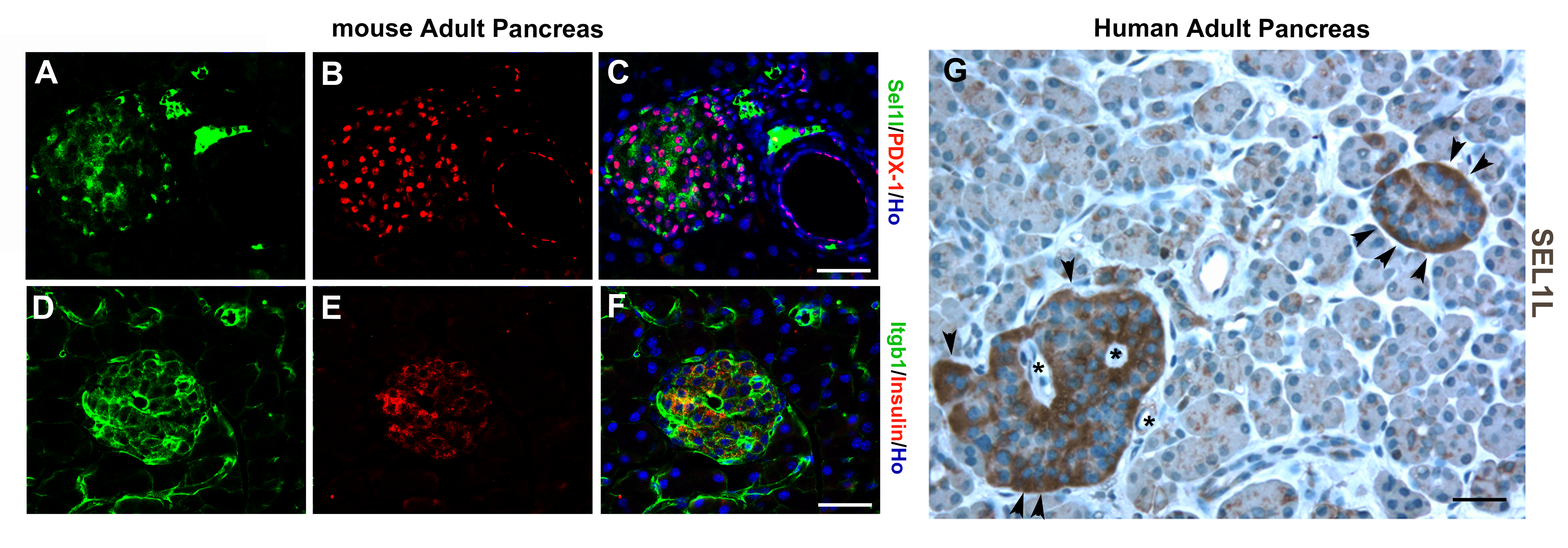

Supplement: Figure S1 — (DOCX) [file pone.0079458.s001.docx]
